# Foliar Abscisic Acid-To-Ethylene Accumulation and Response Regulate Shoot Growth Sensitivity to Mild Drought in Wheat

**DOI:** 10.3389/fpls.2016.00461

**Published:** 2016-04-18

**Authors:** Ravi Valluru, William J. Davies, Matthew P. Reynolds, Ian C. Dodd

**Affiliations:** ^1^Global Wheat Program, International Maize and Wheat Improvement Center (CIMMYT)El Batan, Mexico; ^2^Plant Biology Department, Lancaster Environmental Center, Lancaster UniversityLancaster, UK

**Keywords:** abscisic acid, drought, ethylene, growth sensitivity, hormonal ratio, mild drought

## Abstract

Although, plant hormones play an important role in adjusting growth in response to environmental perturbation, the relative contributions of abscisic acid (ABA) and ethylene remain elusive. Using six spring wheat genotypes differing for stress tolerance, we show that young seedlings of the drought-tolerant (DT) group maintained or increased shoot dry weight (SDW) while the drought-susceptible (DS) group decreased SDW in response to mild drought. Both the DT and DS groups increased endogenous ABA and ethylene concentrations under mild drought compared to control. The DT and DS groups exhibited different SDW response trends, whereby the DS group decreased while the DT group increased SDW, to increased concentrations of ABA and ethylene under mild drought, although both groups decreased ABA/ethylene ratio under mild drought albeit at different levels. We concluded that SDW of the DT and DS groups might be distinctly regulated by specific ABA:ethylene ratio. Further, a foliar-spray of low concentrations (0.1 μM) of ABA increased shoot relative growth rate (RGR) in the DS group while ACC (1-aminocyclopropane-1-carboxylic acid, ethylene precursor) spray increased RGR in both groups compared to control. Furthermore, the DT group accumulated a significantly higher galactose while a significantly lower maltose in the shoot compared to the DS group. Taken all together, these results suggest an impact of ABA, ethylene, and ABA:ethylene ratio on SDW of wheat seedlings that may partly underlie a genotypic variability of different shoot growth sensitivities to drought among crop species under field conditions. We propose that phenotyping based on hormone accumulation, response and hormonal ratio would be a viable, rapid, and an early–stage selection tool aiding genotype selection for stress tolerance.

## Introduction

Drought is a major abiotic stress limiting plant growth and yield. While plant responses differ with drought intensity, timing, and duration (Claeys and Inzé, 2013), we now understand that traits that confer survival of severe stress episodes will not deliver sustained growth and yield under mild stress (Skirycz et al., 2011). From an agricultural viewpoint, severe growth reduction may result in significant yield loss even under mildly stressed field conditions. Most crop species exhibit a large genetic variability of expansion growth (Pereyra-Irujo et al., 2008; Parent et al., 2010; Welcker et al., 2011; Tardieu et al., 2014) and biomass growth (Wang et al., 2008; Boutraa et al., 2010; González, 2011) in response to drought. Therefore, identifying genotypes that maintain, or at least limit the reduction of, growth under stress might be a useful strategy to boost plant biomass (Skirycz et al., 2011; Hatier et al., 2014). Efficient translation of biomass into grains would also enhance yield productivity under stress conditions.

Plants have evolved some adaptive strategies to cope with mild and severe restriction in water availability (Davies and Zhang, 1991). In showing selectivity over the maintenance of either water balance and/or gas exchange, plant species favor either “survival” or “growth” behavior, respectively, when they encounter stress conditions (Tardieu et al., 2014). The latter strategy, which is an opportunistic risk-taking, is generally regarded as a stress-resistance trait (Sade et al., 2012) and plants with this behavior tend to occupy more mild to moderate drought-prone natural habitats (McDowell et al., 2008). This behavior may allow vegetative and reproductive growth under mild to moderate stress conditions but will confer no benefit under conditions of prolonged and severe stress in which plants with the former strategy may survive (Tardieu et al., 2014) but yield can be minimal. Hence, a survival vs. growth strategy of plants differs according to soil moisture. A homeostatic hydraulic regulation is known to partly drive this species specificity (Meinzer et al., 2014); however, some species, grapevine (Chaves et al., 2010), and poplar (Almeida-Rodriguez et al., 2010), can switch between “survival-growth” strategies in response to fluctuating soil moisture. The mechanistic basis of such a dual growth habit is yet to be fully understood, however, it could be regulated by an interaction of hydraulic and chemical signaling.

When drought stress develops, not all leaves respond similarly in stomatal closure (Blum, 2011). It was recently argued that drought insensitive stomata may favor carbon gain at the expense of expansive growth (Caldeira et al., 2014; Tardieu et al., 2014). Biomass accumulation and expansive growth may be controlled by independent environmental and genetic factors (Fatichi et al., 2014) and may govern yield under stress. The positive effect could be through enhancing carbon acquisition, in addition to specific adaptations that allow continued growth under drought (e.g., reprogrammed energy metabolism, osmotic adjustment and high cell wall extensibility; Claeys and Inzé, 2013). The negative effect of wide stomatal aperture on expansion growth could be a consequence of lower hydraulic conductivity (Caldeira et al., 2014). Though the role of abscisic acid (ABA) in plant hydraulics has been debated (Dodd, 2013), ABA can regulate hydraulic conductance (Jia and Davies, 2007; Pantin et al., 2012) via regulation of aquaporins (Sade et al., 2009; Prado et al., 2013). In addition, ethylene, under flooding, can promote (Kamaluddin and Zwiazek, 2002) or inhibit (Li et al., 2009) hydraulic conductivity under phosphorus deficiency depending on the environmental conditions. In addition, auxins and cytokinins closely regulate hydraulic conductivity, and thereby shoot growth, under stress conditions. The interaction between hydraulic and hormonal traits may therefore deliver differences in growth and yielding of crops under drought. We hypothesize that the subtle sensitivity of stomatal and growth traits to chemical regulators can be viewed as a model for species survival–growth behavioral plasticity (Soar et al., 2006; Rogiers et al., 2012) especially under drought.

Plant hormones are well-known to act as growth regulators and their concentrations change in response to numerous stresses (Hays et al., 2007; Ji et al., 2011). Both ABA and ethylene have been shown to exert dual effect on growth: stimulatory at low concentration (Ku et al., 1970; Suge, 1971; Nishizawa and Suge, 1995a,b; Lehman et al., 1996; Smalle et al., 1997) while inhibitory at high (Pratt and Goeschl, 1969; Guzmán and Ecker, 1990; Kieber et al., 1993; Tanaka et al., 2013), a “dose-growth” response phenomenon known as “hormesis” (Pierik et al., 2006; Gressel and Dodds, 2013). Relatively few studies have examined the stimulatory properties of low concentrations of ABA and ethylene (Suge, 1971; Takahashi, 1972; Neskovic et al., 1977; Pierik et al., 2006), suggesting that low concentrations (≤ 0.1 μl L^−1^ or ≤ 0.1 μM) of ethylene and ABA stimulate organ growth to the extent that, across planta, varies widely (0% to >100%) depending on the timing of application, level of organization (e.g., cell, organ), plant species, seedling age, and the physiological and growth conditions. The mechanisms controlling hormone dose-dependent growth response are largely unexplored. Nevertheless, hormetic growth response in general has been vigorously debated in ecotoxicology and medicine and its potential for increasing plant productivity has recently been discussed (Pierik et al., 2006; Gressel and Dodds, 2013).

In this study, we hypothesize that different genotypes may exhibit differential growth sensitivity to drought stress particularly via hormone responses that are normally induced by numerous stresses (Hays et al., 2007; Ji et al., 2011). We show that six spring wheat genotypes differing for stress-susceptibility (see below) exhibit a large genetic variability for early-stage growth sensitivity to very low concentrations of exogenous ABA and ethylene which reflects the yield performance of the genotype under mild stress and/or may indicate more general genotype-specific hormone responses that can benefit growth and yield later in plant development. Further, we show that drought-tolerant and drought-susceptible genotypes differ in their ABA and ethylene accumulation, which might be most likely to occur under mild drought-stressed natural habitats.

## Materials and methods

### Plant material and growth conditions

**Figure 1:**
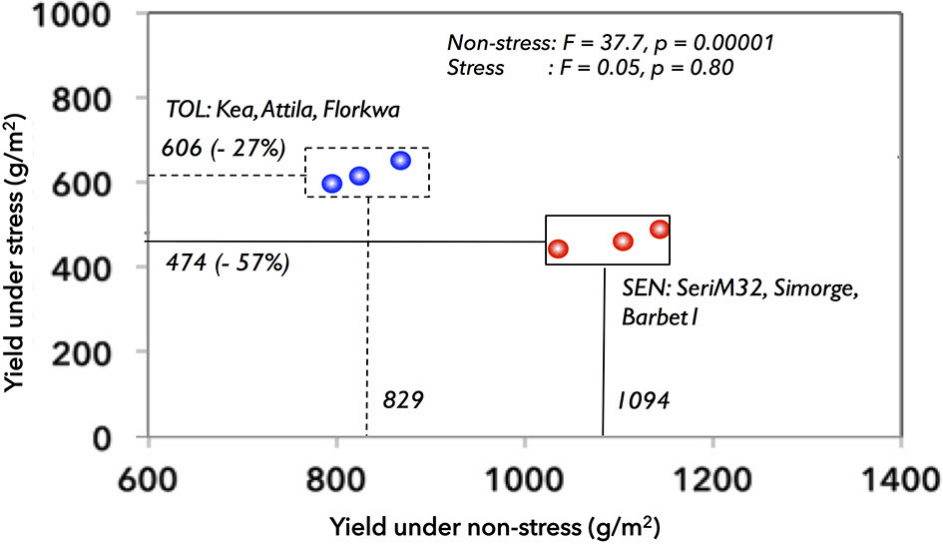
**Grain yields of six different wheat genotypes grown under non-stress and stress conditions in the field**. The selected wheat genotypes were: Tolerant: Kea, Attila, Florkwa; Sensitive: Simorge; Barbeti; SeriM82. SEN, sensitive group; TOL, tolerant group.

Six spring wheat (*Triticum aestivum* L.) genotypes were selected and designated as drought-tolerant (DT: Kea, Attila, Florkwa) or drought-sensitive (DS: SeriM32, Simorge, Barbet1) groups based on their stress susceptibility, biomass accumulation and yield potential in the field (Lopes et al., 2012). These groups were selected in such a way that both the DT and DS groups show contrasting yield susceptibility to stress and non-stress conditions (Figure 1). The DS group had higher yield under non-stress (1094 g/m^2^) while they maintain only 43% of non-stress yields (474 g/m^2^) under stress conditions. In contrast, DT group had lower grain yield under non-stress (829 g/m^2^) as compared to DS group but they maintain 73% (606 g/m^2^) of non-stress yields under stress conditions. Therefore, both groups have differential yield susceptibilities to stress and non-stress conditions. This atypical selection is at marked contrast to a widely accepted breeders conception that selection for high yield potential under non-stress conditions has also improved yield under stress especially for mild to moderate drought stress (Araus et al., 2002, 2008; Trethowan et al., 2002; Cattivelli et al., 2008). However, such genotype selection may be useful to understand the underlying mechanisms of growth and yield responses to the environment.

For all experiments, seeds were initially germinated on a wet-filter paper placed in the Petri dish at room temperature, and a 7-day-old seedlings were transplanted into 0.5 L plastic pots containing a well-prepared mixture of a soil-based compost (John Innes No. 2, UK). Plants were initially grown in a naturally lit glasshouse with supplementary artificial lighting of 200 μmol m^−2^ s^−1^ photosynthetically active radiation (PAR), and a photoperiod of 12 h with day/night temperatures of 25/18°C, respectively. When seedlings reached two-leaf stage, plants were shifted to growth cabinets with an average day/night temperature of 25/22°C, 12 h photoperiod with a relative humidity (RH) of 90%. All plants were well-watered daily and half-strength Hoagland nutrient solution was provided on alternative days. In chemical spray experiments, seedlings (two-leaf stage) were shifted to a modified hydroponic system (50 ml tube-system), in which nutrient solution was changed every 2-days and aeration was continuously provided with aquarium air-pump (BOYU, S-4000B, 3.2 L min^−1^).

### Mild drought stress under controlled conditions

Mild water deficit (MWD) was imposed as described previously (Deokar et al., 2011). When seedlings reached three-leaf stage, water was withheld from all pots to initiate a dry-down procedure. Weight of all individual pots was recorded daily in the morning at ~10.30 h to monitor soil moisture content in both treatments. Daily loss of water through evapotranspiration (ET) was calculated as the difference in pot weight on the current day from that of the previous day. After 7–8 days, when the soil moisture content reached target values of approximately 0.44 and 0.33 g per g^−1^ dry soil in WW and MWD treatments, respectively, (Supplementary Figures S1A–C) all plants were watered daily with the amount of water lost through ET of each pot daily. Thus, WW and MWD plants were maintained at ~94 and 70% of field capacity, reflecting a soil matric potential of ~−0.0048 and −0.08 MPa respectively, as determined from a moisture release curve of the same soil type (Dodd et al., 2006). Both treatments were maintained at the targeted soil moisture for 7 days and leaf samples were then collected before watering to determine endogenous ABA and ethylene accumulation. The remainder of the shoot was harvested separately. Shoot dry weight was determined after samples were oven-dried at 80°C for 72 h. Root growth was not measured in the study. Two experiments with four completely randomized replications for each genotype were conducted.

### Determination of endogenous ABA and ethylene

Endogenous ABA concentrations and ethylene evolution were measured in MWD experiment. For ABA determination, leaf tissues (0.2–0.4 g fresh weight) were collected and immediately frozen in liquid nitrogen. Frozen leaf tissue was freeze-dried for 48 h, finely ground and then extracted in distilled deionized water with an extraction ratio of 1:40 (gram dry weight:mL water) at 4°C overnight. ABA concentrations of the extract were determined using a radioimmunoassay as described (Chen et al., 2013).

Endogenous ethylene emission from leaves was measured using a commercial laser-based ethylene detector (ETD-300, Sensor Sense B.V., Nijmegen, The Netherlands) in combination with a gas handling system (VC-6, Sensor Sense B.V.) as described previously (Wang et al., 2013). Leaf tissues (0.25–0.45 g fresh weight) of WW and MWD plants were sampled, weighed immediately, and placed in 50 mL glass tubes containing moistened filter paper and were allowed wound-induced ethylene to subside (Yang et al., 2006). Later, glass tubes were tightly capped with a double-bent rubber stopper and were further incubated for 5 h in the light at the room temperature. Using a 5-mL syringe, 4 mL gas was extracted through rubber stopper, and stored in 4 mL sealed glass vials. These vials were connected to inlet and outlet cuvettes of VC-6 system, which allow six cuvettes at once, and continuously flushed with air at a constant flow of 4 L h^−1^. Ethylene emission from each vial was monitored alternatively by ethylene detector in sample-mode for 10 min. To remove any traces of external ethylene or other hydrocarbons, the airflow was passed through a platinum-based catalyser before entering the cuvettes. A scrubber with KOH and CaCl_2_ was placed before ethylene detector to reduce the CO_2_ and water content in the gas flow, respectively. The ethylene emission was corrected for tissue fresh weight and the duration of incubation to determine ethylene emission rate.

### Drought trials under field conditions

Four field trials were conducted during 2009–10 and 2010–11 under two different growth environments: two under well-irrigated conditions (controls, total crop water supply >700 mm), and two under drought (total crop water supply ≤ 300 mm). All trials were sown in alpha-lattice design with two replicates in the Yaqui Valley at CIMMYT's Obregon Experimental Station in North-Western Mexico (27°25′N 109°54′W, 38 m above sea level). Detailed trial procedures and meteorological data were described elsewhere (Lopes et al., 2012, 2015; Sukumaran et al., 2015). Briefly, the sowings were made in late November each year with either irrigation or drought. In drought trials, irrigation was at sowing with no further irrigation, making ~180 mm of water available to the crop. The experimental design was a randomized lattice with two replications in 2 m long and 0.8 m wide plots consisting of one raised bed with two rows per bed at seed rate of 120 kg/ha. Appropriate weed, fertilization, disease, and pest control were followed to avoid any yield limitations. When seedlings were at three-leaf stage (~23 days after sowing), normalized difference vegetation index (NDVI, a proxy for biomass) was measured along the length of the plot but avoided the boarders (0.25 m each side), averaged across trials and years and mean values were presented.

### ABA and ACC spray experiment

When the seedlings reached three-fully emerged leaves, plants were foliar-sprayed with ABA and ACC (1-aminocyclopropane-1-carboxylic acid, ethylene precursor) as described previously (Chen et al., 2013). The optimal concentrations (at which shoot growth response is maximal) of ABA and ACC concentrations were determined in preliminary experiments (Supplementary Figure S1D). ACC, the endogenous ethylene precursor, was preferred as a source of ethylene to ethephon (a phosphonic acid), since non-ethylene generating phosphonic acids can have physiological effects on plants (Ernst et al., 1992; Chen et al., 2013). ACC was dissolved in water while ABA was dissolved in ethanol for stock solution preparation and a wetting agent Silwet (L-77, De Sangosse Ltd, Cambridge, UK) at 0.025% (v/v) was included in all solutions. Two-hours into the photoperiod, plants were foliar-sprayed (4-5 mL plant^−1^) either with water that contain ethanol and Silwet (controls), ABA (0.1 μM), or ACC (0.1 μM) assuming that a proportion of each chemical sprayed onto leaf surface will penetrate the leaf interior (Wilkinson and Davies, 2008). After spraying, plants were grown further for 7 days in the same hydroponic system and then harvested to determine shoot fresh weight. Shoot dry weight was determined after oven-drying at 80°C for 72 h. Shoot dry weight at the beginning (just before spray) and end of (7-days) treatment were used to calculate relative shoot growth rate (RGR) according to (Hoffmann and Poorter, 2002). The experiment was repeated twice, with four completely randomized replications for each genotype. We also measured RGR at six-leaf stage whereby plants were foliar-sprayed with ABA and ACC at the same concentration (0.1 μM) at the three-leaf stage.

### Determination of carbohydrates

In the chemical spray experiment, sugars, and sugar alcohols (sucrose, glucose, fructose, raffinose, erlose, maltose, galactose, rhamnose, sorbitol) of the leaf tissue were quantified using high performance liquid chromatography (HPLC) method as described previously (O'Rourke et al., 2015). Grounded dry tissue samples (20–50 mg) were extracted two-times with 2.5 ml of 80% ethanol by boiling the samples in glass tubes in a 60°C water bath for 30 min each. After each extraction, the tubes were centrifuged at 4500 rpm for 10 min, and the extracts were then pooled and dried in a speedvac for ~3–4 h. From this, final extract 200 μL was further dried down to remove the ethanol and rediluted with 200 μL deionized water. HPLC with a Dionex IC-3000 system including electrochemical detection cell with gold electrode and temperature controlled column compartment at 30°C (Thermo Scientific, Hemel Hempsted, UK) was used. The column used was a Dionex CarboPac PA20 3 × 150 mm analytical column (Thermo Scientific, Hemel Hempsted, UK). Ten microliters of sample was injected into the sample loop connected to the ion exchange column. The peaks were identified by comparing retention times with those of standard sugar markers with Dionex Chromeleon software.

### Data analyses

Statistical analyses were performed with R 3.0.1 (R Development Core Team, 2013). Data were averaged across genotypes, groups and treatments and mean values were reported. Two-way ANOVA considered treatments (WW and MWD) and groups (DT and DS) as explanatory variables while shoot dry weight was a response variable. An ANCOVA model was used considering SDW as the dependent variable with groups as the factor and hormones as the covariates. Principal component analysis (PCA) was performed on carbohydrate data to identify the carbohydrate response patterns between DT and DS groups as well as between the treatments. One-way ANOVA was used for the effects of exogenous hormones on the shoot growth rate, carbohydrates, and a Student's *t*-test was used to compute the pair-wise comparisons between group means with Bonferroni correction.

## Results

### Drought-tolerant and drought-sensitive genotypes show different shoot growth responses to mild drought

**Figure 2:**
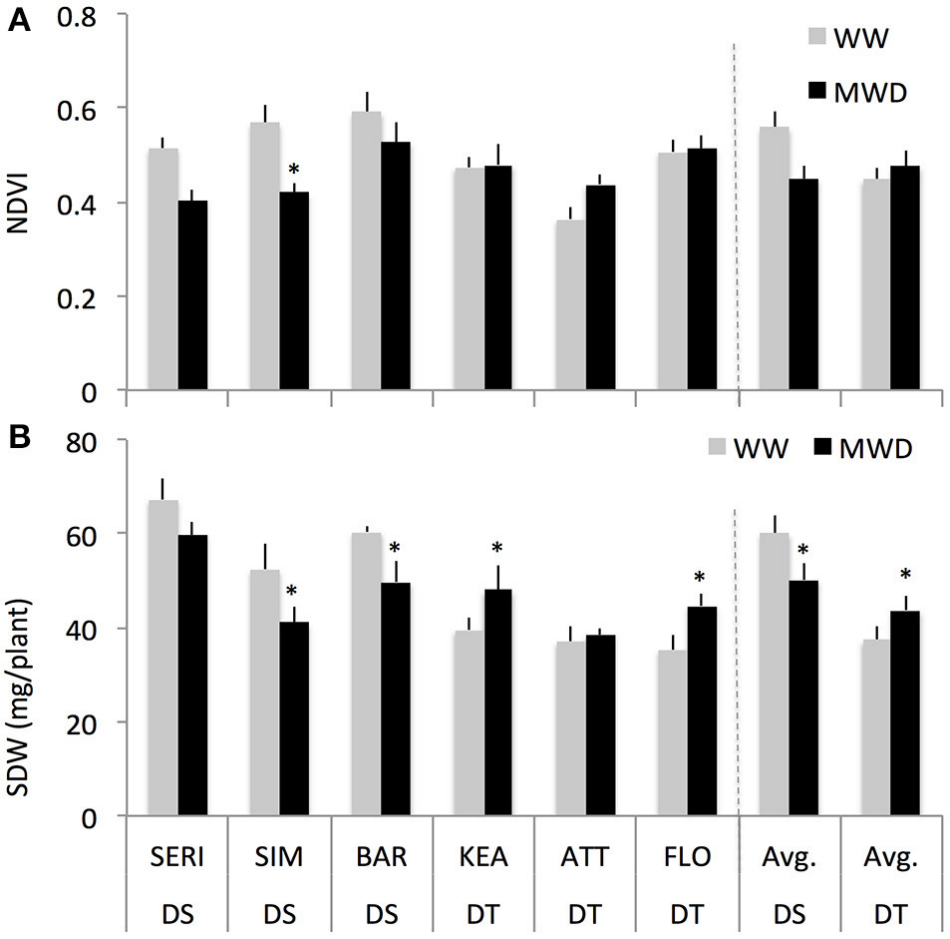
**The normalized difference vegetation index (NDVI) values (A) and shoot dry weight [SDW, (B)] of drought-tolerant (DT) and drought-susceptible (DS) genotypes at 3rd leaf stage grown under field-drought (with two replications) and controlled drought conditions with four replications for each genotype, respectively**. ^*^ indicates *p* < 0.05.

We studied the effect of mild drought on shoot growth response at the three-leaf stage of six wheat genotypes differing for drought sensitivity. Across all genotypes, average NDVI values measured at the three-leaf stage (23 DAS) in the field were comparable between WW and WD conditions (data not shown). When all genotypes were separated into drought-tolerant (DT, 3) and drought-sensitive (DS, 3) groups, the DS group showed a higher NDVI (13%) compared to DT group in WW conditions (Figure 2A). However, under mild drought, the DS group showed a reduction in NDVI (−7%) while the DT group had a slightly increased NDVI (+0.6%). Such NDVI responses were not significantly different between the DT and DS groups.

Consistent with the NDVI results (Figure 2A), both the DT and DS groups showed similar shoot dry weight (SDW) responses under controlled mild drought conditions albeit with a greater relative response (Figure 2B). The DS group had higher SDW (60%) than DT group under WW conditions. Under MWD, the DS group however showed a reduction in SDW (–16%) while the DT group had an increased SDW (+17%) relative to WW plants of the same group. A two-way ANOVA indicates that there was a significant interaction effect of groups and treatments on SDW (*P* = 0.008; for groups: *P* < 0.001; for treatments: *P* = 0.05). Further, treatments have significant effect on SDW within DS (*P* = 0.03) and DT (*P* = 0.03) groups. Similar shoot fresh weight responses of DT and DS groups in WW and MWD conditions were observed (Supplementary Figure S2A).

We examined whether differences in SDW of DT and DS groups could be related to plant water content. This seems unlikely, as both groups showed a tight association between FW and DW in both the conditions (Supplementary Figure S2B). DS plants have slightly more water content (< 1%) but both groups responded similarly to MWD (Supplementary Figure S2C). Overall, these results suggest that the two groups of wheat cultivars responded differently to mild drought.

### Shoot growth sensitivity of drought-tolerant and drought-sensitive groups was closely associated with endogenous ABA and ethylene accumulation and responses

**Figure 3:**
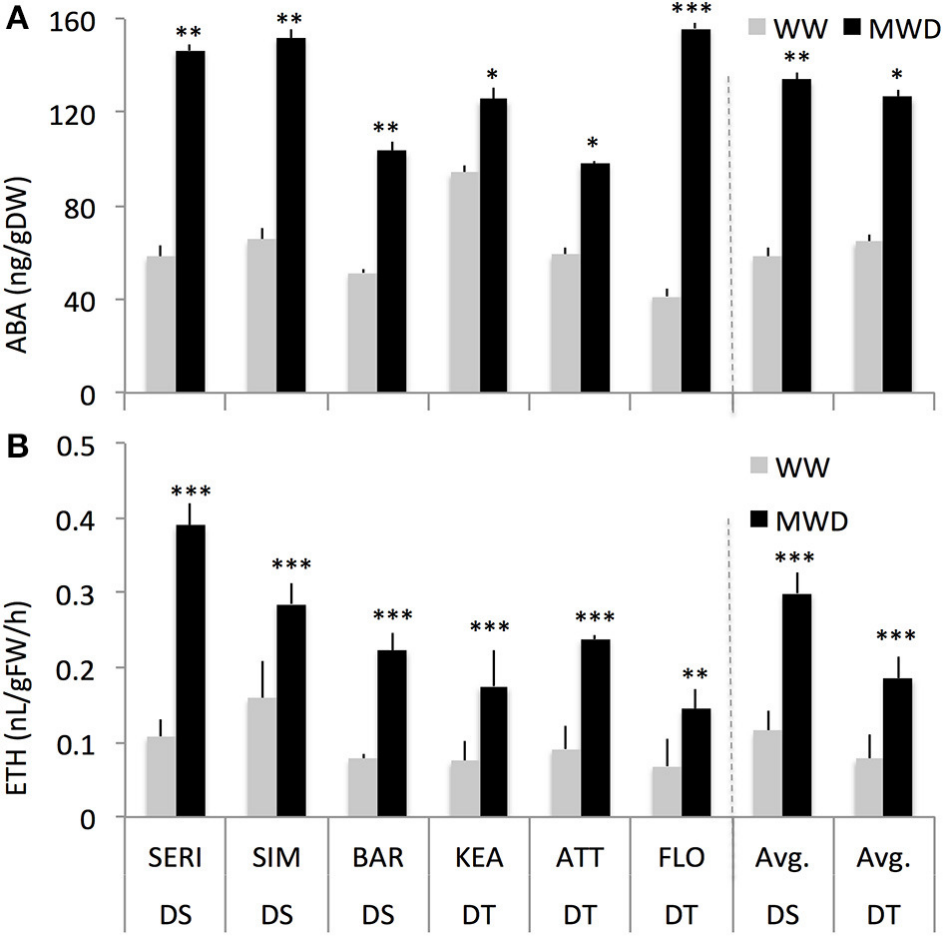
**Shoot ABA concentration (A) and ethylene production (B) of drought-tolerant (DT) and drought-susceptible (DS) genotypes grown under well-watered (WW) or mild water-deficit (MWD) conditions with four replications for each genotype**. ^*^, ^**^, ^***^ Indicate *p* < 0.001, < 0.01, and < 0.05, respectively.

Previous studies have reported that wheat genotypes differ in the accumulation of, and their sensitivity to, ABA (Ji et al., 2011). We, therefore, measured endogenous ABA and ethylene in DT and DS genotypes grown under WW and MWD conditions. The DS and DT groups showed similar pattern of ABA and ethylene accumulation with both groups showing significantly higher ABA concentration (129% and 95%, respectively; *P* < 0.001) and ethylene production (160 and 138%, respectively; *P* = 0.001) in response to MWD (Figure 3, Supplementary Figures S3A,B). The main group effect on ABA was not significant (*P* > 0.05) but was significant for ethylene production (*P* = 0.01), indicating that both groups (DT and DS) significantly differed for ethylene production.

**Figure 4:**
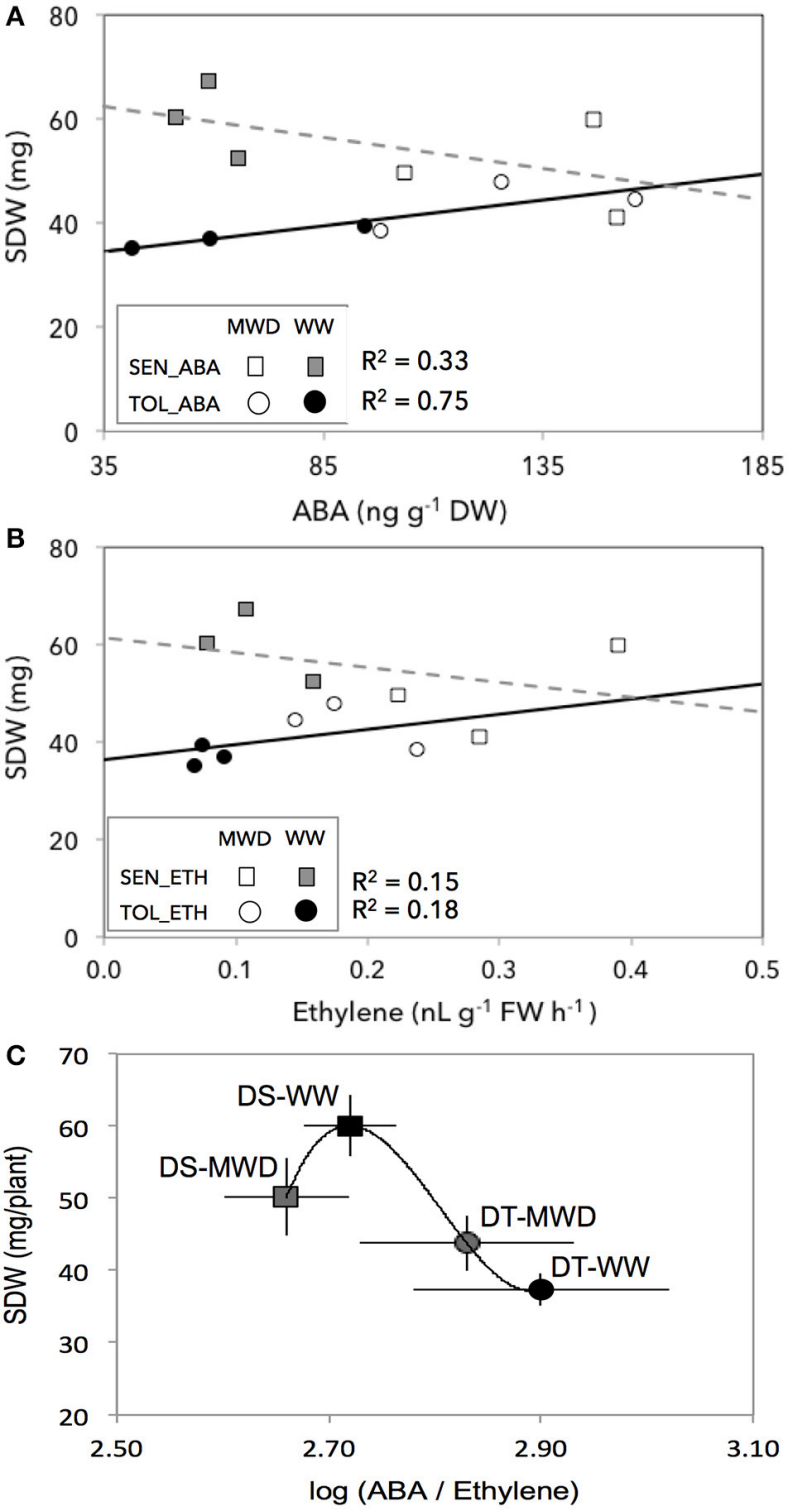
**The association between shoot dry weight (SDW) and endogenous ABA (A), ethylene (B), and ABA:ethylene ratio (C) in drought-tolerant (circles, DT) and drought-susceptible (squares, DS) genotypes grown under well-watered (filled symbols) and mild drought (open symbols) with four replications for each genotype**. SEN, drought-susceptible; TOL, drought-tolerant. ETH, ethylene; ABA, abscisic acid.

Across two treatments, SDW responses of the DT and DS groups to endogenous ABA and ethylene showed a tendency toward two response trends (Figures 4A,B, group effects for ABA: *P* < 0.0001; group effects for ethylene: *P* < 0.0001). The DT group showed an increased SDW with increasing concentrations of ABA (round circles, Figure 4A). In contrast, the DS group showed a decreased SDW with increasing concentrations of ABA (squares, Figure 4A). Such SDW responses of the DT and DS groups were consistent with ethylene whereby both the DT and DS groups showed an increased and a decreased SDW response to increasing levels of ethylene, respectively (Figure 4B). Such differential SDW response trends between DT and DS groups were largely driven by WW conditions, suggesting that hormone concentrations may regulate shoot growth even under optimal growing conditions.

Across treatments (WW and MWD) and groups (DT and DS), SDW did not correlate with ABA (*r*^2^ = 0.003, *P* = 0.81; Supplementary Figure S3C) and ethylene (*r*^2^ = 0.15, *P* = 0.12; Supplementary Figure S3D). However, SDW responses of both groups followed ABA:ethylene ratio (Figure 4C) that fits well with their SDW responses to mild drought (Figure 2B). Among the four-subgroups (DS-WW, DS-MWD, DT-WW, and DT-MWD), DS-WW subgroup had a higher SDW with an ABA/ethylene ratio of 2.72 while DT-WW subgroup had a lower SDW with an ABA/ethylene ratio of 2.90. However, both groups reduced ABA:ethylene ratio in response to MWD albeit at different level (2.66 and 2.83, respectively) but were not significantly different between two groups. These results suggest that an appropriate ABA:ethylene ratio might be critical and the DT and DS groups exhibited a differential growth sensitivity to MWD by differential accumulation of ABA and ethylene.

### Foliar-spray of exogenous ABA and ACC increase shoot relative growth rate of drought-tolerant and drought-sensitive groups under well-watered condition

**Figure 5:**
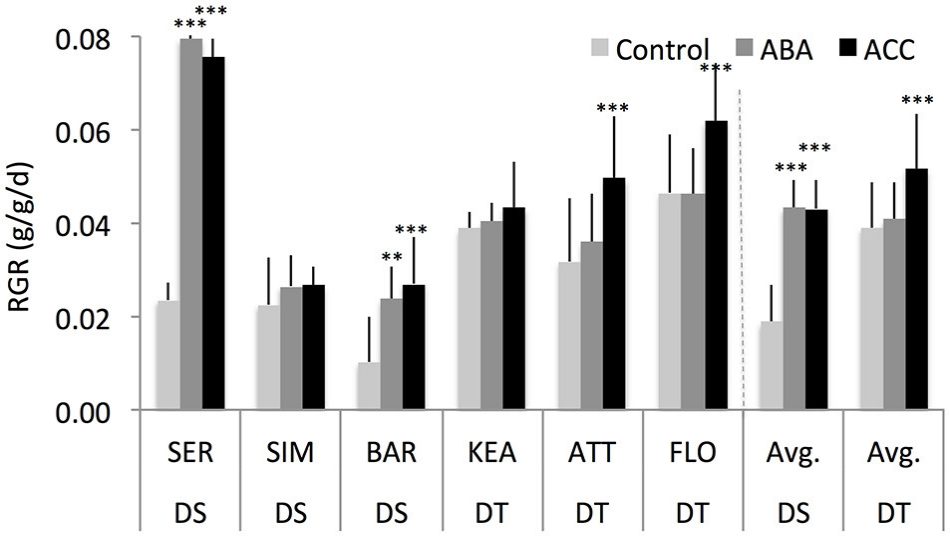
**Shoot relative growth rate (RGR) of six wheat genotypes at 3rd leaf stage that were either sprayed with water (controls), abscisic acid (ABA, 0.1 μM), or the ethylene-precursor, 1-aminocyclopropane-1-carboxylic acid (ACC, 0.1 μM) with four replications for each genotype**. ^**^, ^***^ indicate *p* < 0.01, and < 0.05, respectively.

Previous studies have often shown that very mild concentrations of exogenous ABA (Takahashi, 1972; Watanabe and Takahashi, 1997) and ethylene (Burg and Burg, 1966, 1968) stimulated growth of various organs of a range of plant species. We, therefore, examined whether low concentrations of exogenous ABA and ethylene could stimulate growth of DT and DS genotypes under WW condition. Both the DT and DS groups showed a significantly different shoot relative growth rate (RGR) response to exogenous ABA and ACC spray (Figure 5). ABA and ACC strongly promoted RGR of DS genotypes (131 and 130% respectively; *P* = 0.01) but had modest effect on RGR of DT genotypes (5 and 32% respectively; *P* = 0.03). Across all groups, ABA had an increased RGR by 50% (*P* = 0.04), while ACC was slightly more effective in stimulating shoot RGR (78%, *P* = 0.002). Such growth stimulation responses to exogenous ABA and ACC sprayed at the three-leaf stage were not significantly different from control at the 6th leaf-stage (Supplementary Figure S4), suggesting that low concentrations of ABA and ACC stimulated growth response may be dependent on the developmental stages.

### Foliar-spray of exogenous ABA and ACC had differential effects on carbohydrates status of drought-tolerant and drought-sensitive groups under well-watered condition

We hypothesized that an increased RGR response to low concentrations of ABA and ACC could be related to an altered carbohydrate status in DT and DS groups. A two-way ANOVA indicates that there was a significant interaction effect of groups and treatments for carbohydrates such as rhamnose (*P* = 0.025), raffinose (*P* = 0.003), and maltose (*P* = 0.000) (Supplementary Table S1). Further, treatments and groups have significant effect on galactose (*P* = 0.000 and 0.000, respectively), glucose (*P* = 0.000 and 0.000, respectively), fructose (*P* = 0.004 and 0.054, respectively; group has marginal effect on fructose), and maltose (*P* = 0.004 and 0.000, respectively). However, there was no interaction effect of treatment and group on these carbohydrates (Supplementary Table S1).

**Figure 6:**
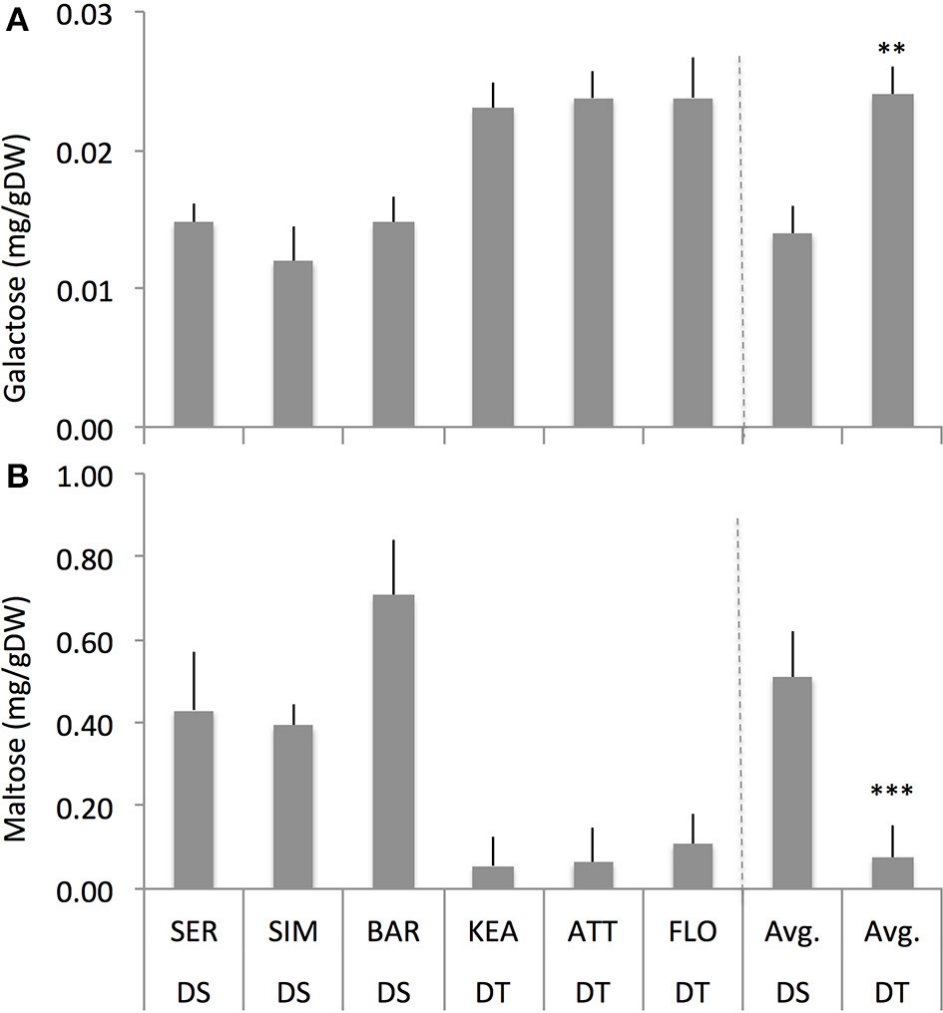
**The concentrations of galactose (A) and maltose (B) of drought-susceptible (DS) and drought-tolerant (DT) genotypes across the treatments (control, ABA and ACC (1-aminocyclopropane-1-carboxylic acid) spray) with four replications for each genotype**. ^**^, ^***^ indicate *p* < 0.01, and < 0.05, respectively.

**Figure 7:**
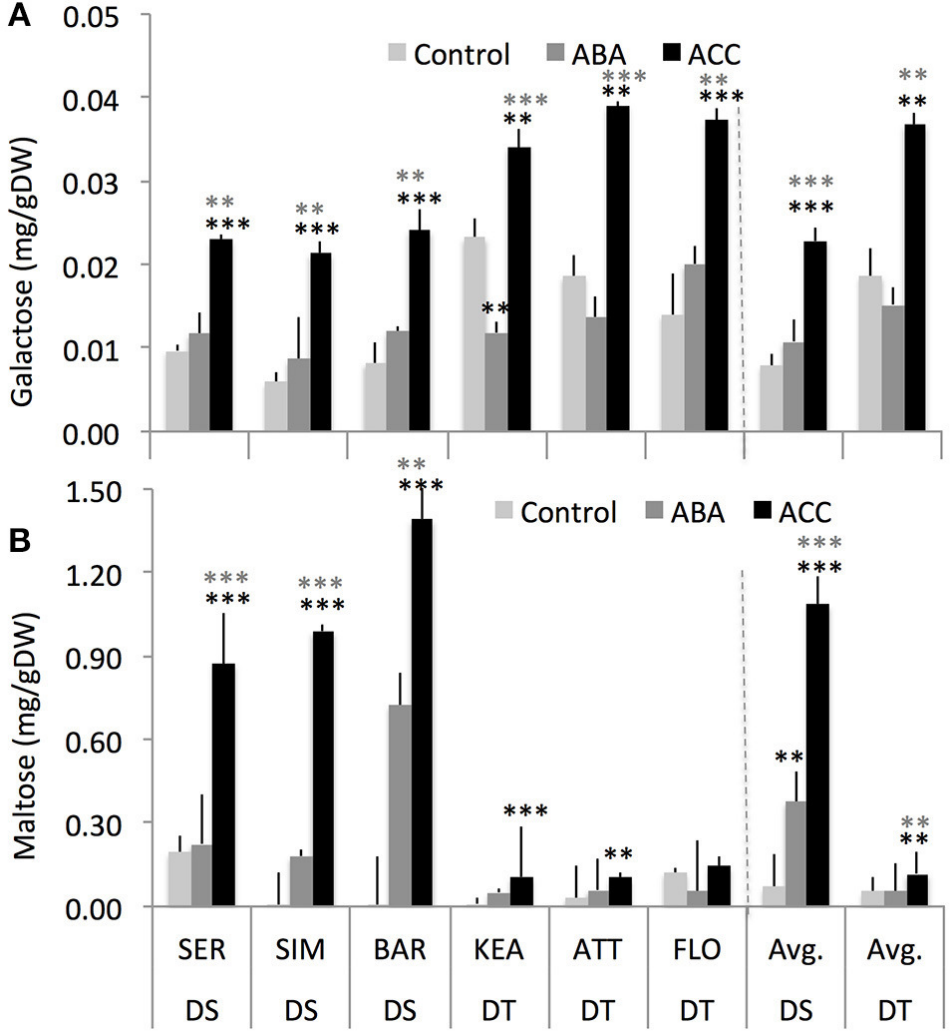
**The effect of chemical spay on the concentrations of galactose (A), and maltose (B) between drought-susceptible (DS) and drought-tolerant (DT) genotypes at 3rd leaf stage that were either sprayed with water (controls), abscisic acid (ABA, 0.1 μM), or the ethylene-precursor, 1-aminocyclopropane-1-carboxylic acid (ACC, 0.1 μM) with four replications for each genotype**. ^**^, ^***^ indicate *p* < 0.01, and < 0.05, respectively.

Across two treatments, DS group had significantly lower galactose (–45%, *P* = 0.008) but had significantly higher maltose (+575%, *P* = 0.000) compared to the DT group (Figure 6) while both DS and DT groups do not show significant difference for sucrose, fructose, rhamnose, raffinose, erlose, and sorbitol (Supplementary Figure S5). Among the treatments, ACC had consistently significant effect on galactose (*P* = 0.000 and 0.001), and maltose (*P* = 0.000 and 0.025) in both the DS and DT groups, respectively (Figure 7). Although ABA increased these carbohydrates as well, it had significant effect only for maltose (*P* = 0.023) in DS group but not in DT group. Overall, these results indicate that both the DT and DS groups had altered carbohydrates status in response to foliar ABA and ACC spray (Figures 6, 7).

## Discussion

Previous studies on shoot growth plasticity to varying soil moisture have provided valuable information leading to current understanding of growth control by multiple processes. We have further extended our understanding to assess the importance of individual physiological traits in the context of crop growth under stress conditions. First, we propose that a simple shoot biomass growth assay (Figure 2) can be used as a sensitive indicator of stress tolerance (Claeys et al., 2014). Second, an early seedling stage represents a suitable growing tissue for deducing precise drought adaptive mechanisms controlling growth since drought adaptive mechanisms differ between young growing- and mature-tissues (Harb et al., 2010; Skirycz et al., 2010). Third, many QTLs for several seedling-stage traits, including early shoot biomass, co-locate with QTLs linked with grain yields (Sandhu et al., 2015); hence, seedling responses can be relevant for crop yields under field conditions. This study encompasses the first two propositions and further suggests that an early seedling-stage can predict stress-adapted traits and reduces the time needed for genotype selections prior to time-consuming phenotypic evaluations under field conditions.

### Mild drought stress enhanced shoot dry biomass in drought-tolerant genotypes but not in drought-susceptible genotypes

Our study showed that the DT group could enhance, or maintain, shoot biomass growth under mild drought conditions as compared to the DS group (Figures 2A,B). Such differential growth responses between DT and DS groups may be controlled by genotype-specific mechanisms (Hall et al., 1982; Chaves et al., 2002; Campos et al., 2004; An et al., 2014). Supporting, both DT and DS groups exhibited differential hormone responses (Figure 3) and ABA:ethylene ratios (Figure 4) when exposed to mild drought. Though marginally significant (*P* = 0.056), the DT group had a higher *g*_*s*_ under MWD compared to WW conditions (Supplementary Figure S2D), suggesting a sustained *g*_*s*_ may enhance carbon gain (Caldeira et al., 2014; Tardieu et al., 2014), and subsequently crop yield (Fischer et al., 1998; Lu et al., 1998), under mild stress conditions. Such differential stomatal responses between DT (small stomatal responses) and DS (large stomatal responses) genotypes have previously been reported and have also been shown to correlate with yield under stress conditions (Tardieu and Simonneau, 1998; Munns et al., 2010; Sade et al., 2012; Tardieu et al., 2014).

The enhanced shoot growth of DT group under MWD in our study might be related to the fact that we imposed a steady-state MWD on growing tissues of young seedlings, which greatly differ with mature tissues, for stress adaptive mechanisms (Lechner et al., 2008). We further propose that the positive growth response of young growing seedlings, such as in this study, might be easier to detect when not dominated by a negative or no growth response of mature tissues particularly at later developmental stages. While such differential growth responses between young and mature tissues at different developmental stages under MWD is worth for follow-up studied, it may partly explain a widely reported negative growth response of plants under drought that contain proportionately more mature tissue than young growing tissue. Such mechanisms may differ between the DT and DS genotypes (Ji et al., 2011).

Our results agree with previous studies that have reported enhanced shoot dry biomass under mild drought (Liu and Li, 2005; Boutraa et al., 2010). Additional evidence that mild-stresses can enhance shoot biomass growth comes from studies with two indica rice cultivars, where mild-salt stress (NaCl at 0.5% w/v) increased shoot dry weight in 4-weeks after treatment (Sripinyowanich et al., 2013; Tada et al., 2014). Taken all together, we postulate that mild stresses may enhance biomass growth at least in stress-tolerant genotypes although the precise underlying mechanisms can be debatable. Higher *g*_*s*_ can be an obvious important stress-associated trait that can contribute to an increased carbon (C) gain in the DT genotypes during an initial stages of drought stress (Caldeira et al., 2014; Tardieu et al., 2014). In addition, mechanisms that involve lower energy costs, for example, lower root respiration could be important for growth regulation under MWD, as reported in drought-tolerant wheat genotype (Liu and Li, 2005).

### ABA:ethylene ratio is an important trait in shoot growth regulation under mild drought that differs between drought-tolerant and drought-susceptible genotypes

It has long been known that plant hormones form a complex network to coordinate the regulation of numerous development processes. ABA and ethylene interactions in regulating numerous biological processes have been well-reported at the cell level (Tanaka et al., 2005; Beguerisse-Dıaz et al., 2012; Chen et al., 2013; Watkins et al., 2014). However, although the chemical control of growth by these hormones has been demonstrated in specific tissues (Sharp and LeNoble, 2002), our eco-physiological understanding of the regulation of these hormones in field crops at the whole plant/crop level is rather limited (Parent et al., 2009; Caldeira et al., 2014; Planes et al., 2015) as these hormones affect a very large number of processes and their interactions are complex. This study suggests a key role for an optimum threshold of ABA:ethylene ratio in regulating shoot biomass growth (Figure 4) whereby both the DT and DS groups had different ABA:ethylene ratios in response to mild drought. Such an ABA:ethylene ratio might be different for other crop species and physiological processes studied and may be specific to developmental stages, an issue worthy to be studied. We propose that genotypes and/or environmental conditions that lead to an optimum hormonal ratio under mild stress conditions—as was shown in this study—may allow greater shoot biomass gain as long as the hormonal ratio in other tissues is not detrimental, which may be different under more severe stress.

Our results complement several studies that have previously demonstrated the key roles of hormonal ratio sensing, for example, auxin:cytokinin ratio in shoot, root induction (Skoog and Miller, 1957; Mercier et al., 2003), and shoot vigor (Albacete et al., 2008), cytokinin:auxin ratio in shoot and inflorescence regeneration (Cheng et al., 2010), gibberellin:abscisic acid ratio in barley grains (Weier et al., 2014), and arabidopsis seed development (Yamaguchi, 2008), most likely through differential gene expressions (Weier et al., 2014). Knowing that ethylene is neither actively transported nor degraded, although ACC oxidase activity is constitutively present in most vegetative plant tissues, and that both DT and DS groups significantly differed for ethylene accumulation but not for ABA, strongly suggest that genetic variability in ethylene biosynthesis may play a crucial role in the changes of ABA:ethylene ratio and its effect on shoot dry biomass of plants. This is further supported by the fact that, across two groups, ethylene was increased by 149% while ABA was increased by 112% under mild drought, not inconsistent with previous studies reporting a higher (five-fold) and lower (two-five-fold) increases for ethylene and ABA, respectively, in a salt-stressed tomato (Albacete et al., 2008). Indeed, ethylene response transcription factors (ERF5/ERF6) have been proposed to act as molecular nodes in the stress-related network where growth control and stress tolerance diverge (Claeys and Inzé, 2013; Dubois et al., 2013). Because, wheat genotypes exhibit a large genetic variability in biosynthesis of, and sensitivity to, ABA and ethylene (Quarrie and Lister, 1983; Sridhar, 2003; Iehisa and Takumi, 2012; Valluru et al., 2014), a natural variation in ABA and ethylene biosynthesis, and consequently ABA:ethylene ratio, might reflect a genetic determinism that partly drive biomass accumulation among crop species under mild stress conditions. Therefore, hormonal ratio can be an invaluable candidate trait for the selection of genotypes for achieving higher biomass and yield under mild stress conditions (Wilkinson et al., 2012).

While our study emphasizes hormonal ratio influences on both plant growth and functioning, it does not throw light upon the mechanistic basis of the maintenance of an appropriate ABA:ethylene ratio in plants. We, however, propose that such an optimal ABA:ethylene ratio, rather than single hormone level, could be a sensitive regulator (or sensor) of, for instance, appropriate morphological development and physiological functioning (Weier et al., 2014; Zhang et al., 2014) providing a fitness advantage in complex natural environments. Plants may respond to environmental perturbations by synthesizing different hormone levels, thereby different hormonal ratio, enabling the communication and transduction of environmental cues into plastic responses (Pozo et al., 2015). It is now widely accepted that ethylene and ABA interact at multiple levels (Cheng et al., 2009; Krouk et al., 2011) and ABA induced stomatal closure has been widely shown to be antagonized by ethylene. An optimal ABA:ethylene ratio therefore keeps stomata partly open (higher *g*_*s*_) allowing enhanced gas exchange that indeed allow continued C gain in DT genotypes under mild drought. While expansive growth may be directly limited by hydraulic signals (Caldeira et al., 2014), continued C gain is important for attaining dry biomass gain when water status is re-established. However, modifying the hormonal ratio by attaining moderate levels of hormones through breeding remains a major challenge. Exploring phenotypic screens of large numbers of genotypes including landraces, wild relatives (Sridhar, 2003; Iehisa and Takumi, 2012; Valluru et al., 2014) and the use of molecular approaches targeted at specific tissues and growth stages (Habben et al., 2014) would facilitate the development of crop cultivars that are able to grow under numerous abiotic stress conditions with minimal yield losses (Peleg and Blumwald, 2011).

### Low concentrations of ABA and ACC increase shoot relative growth and alter carbohydrate status that differ between drought-tolerant and drought-susceptible genotypes

Generally, the action of ABA and ethylene at the higher concentration has been related with the process of growth inhibition. However, there is recent evidence of their presence in developing tissues and also of being organ/tissue and development stage-specific where they may have a promoting action (Finkelstein and Rock, 2002; Sansberro et al., 2004; Peng et al., 2006; Skirycz et al., 2010; Duan et al., 2013). Our results demonstrate that low concentrations of ABA and ACC sprayed onto the seedlings favored vegetative growth, benefitting dry matter accumulation of wheat seedlings under optimal growing conditions particularly for DS genotypes (Figure 5). These results agree with previous studies that have reported that field-grown wheat plants treated with ABA (300 mg L^−1^) under water stress showed higher shoot biomass accumulation (Travaglia et al., 2007, 2010). Further, exogenous ABA (10 mg L^−1^) application at anthesis stage increased dry matter accumulation 7 days after anthesis in a field-grown stay-green wheat line (Yang et al., 2014). In addition, ABA (300 mg L^−1^) sprayed onto the leaves of soybean plants showed an enhanced dry matter accumulation under field conditions (Travaglia et al., 2009). Moreover, ABA and ACC spray lead to the accumulation of specific carbohydrates in leaves (Figure 7). Overall, these results suggest that both ABA and ethylene at low concentration may be important regulators of shoot biomass likely due to improved physiological parameters such as chlorophyll, green leaf area and duration, photosynthesis, and carbohydrate status (source effects), as reported previously (Khan, 2004; Travaglia et al., 2007, 2010; Khan et al., 2008; Iqbal et al., 2011, 2012).

Again, both DT and DS groups showed differences in the accumulation of specific carbohydrates (Figure 6). The DS group had significantly lower galactose but had significantly higher maltose contents compared to the DT group. This suggest that the DS group had more utilization of sugars such as galactose (galactose is directly converted to glucose, for example, in wheat seedlings; Hassid et al., 1956), and maltose levels (Figure 6) compared to the DT group. Higher maltose levels indicate high turnover of starch. Although galactose at higher concentration has often been shown to be detrimental to organ growth, lower concentrations of galactose can transiently increase the sink demand for carbon, and therefore, enhances carbon unloading from the phloem (Thorpe et al., 1999). Because galactose is known as a unique sugar that increase carbon import and phloem unloading, it may offer avenues to examine possible sugar signals resulting in phloem unloading in sink tissues and consequent biomass development (Seifert et al., 2002) especially when compared the DT and DS genotypes.

In addition to growth stimulation, low-concentrations of ABA and ethylene may condition the crop plants that, in essence, would provide competence for adaptation to stresses of similar or others (Bartels et al., 1990). Since these hormones have knock-on effects on several growth processes that can also be measured, this study therefore suggests that phenotyping for low-concentrations of ABA- and ethylene-induced growth *per se* would potentially represent a positive contribution to crop biomass and yield under field conditions (Figure 5, Cai et al., 2014), and may also lead to novel germplasm being made available to breeders for the development of high yielding and stress adapted crop cultivars.

In conclusion, the hormone interaction presented here may deliver benefits in terms of dry biomass gain under mild stress conditions. In environments with optimal to sub-optimal growing conditions, which induce slightly elevated concentrations of both hormones, the ABA and ethylene ratio presented here may underlie a part of genetic determinism that control shoot dry biomass gain in wheat. This is supported by our results that (1) both the DT and DS groups exhibited different SDW responses to mild drought (Figure 2; Liu and Li, 2005; Boutraa et al., 2010), (2) mild drought induced low concentrations of ABA and ethylene (Figure 3 Wright, 1977; Ali et al., 1999; Dodd et al., 2010), and (3) low concentrations of ABA and ACC stimulated SDW of wheat seedlings (Figure 5; Takahashi, 1972; Watanabe and Takahashi, 1997) likely through altered carbohydrates status of the plants (Figures 6, 7).

## Author contributions

RV, WD: Designed, conducted and oversee the glasshouse experiments; MR: designed and oversee the field experiments; RV, WD, MR and ID: wrote the paper. All authors read and approved the final manuscript.

### Conflict of interest statement

The authors declare that the research was conducted in the absence of any commercial or financial relationships that could be construed as a potential conflict of interest.

## Supplementary material

Supplementary Figure S1 **(A),** Soil moisture content (g H_2_O g^−1^ dry soil) in both well-watered and mild drought stress treatments; **(B)**, Soil water potential (MPa) of both well-watered and mild-drought stressed plants; **(C)**, Temperature and humidity levels during the experiment, and **(D)**, Primary leaf growth response to different concentrations of ABA and ACC.

Supplementary Figure S2 **(A)** Shoot fresh weight of six wheat genotypes that were either grown under well-watered (WW) or mild water-deficit (MWD) conditions. **(B)**, the relationship between log values of fresh weight (FW) and dry weight (DW) in WW and MWD treatments; **(C)**, plant water content (%) of drought-susceptible and drought-tolerant wheat genotypes in WW and MWD treatments; **(D)**, stomatal conductance of drought-susceptible and drought-tolerant wheat genotypes in WW and MWD treatments.

Supplementary Figure S3 **ABA concentration (A) and ethylene evolution (B) in well-watered (WW) and mild-drought stress (MWD) treatments across all genotypes**. Linear correlations between shoot dry weight (SDW) and endogenous ABA **(C)** and ethylene **(D)** across all groups and treatments. ^**^ indicates *p* < 0.01.

Supplementary Figure S4 **Shoot relative growth rate in drought-susceptible (A) and drought-tolerant (B) wheat genotypes that were either sprayed with water (controls), abscisic acid (ABA, 0.1 μM) or the ethylene-precursor, 1-aminocyclopropane-1-carboxylic acid (ACC, 0.1 μM) at the 6th leaf stage**.

Supplementary Figure S5 **Sucrose (A), fructose (B), rhamnose (C), raffinose (D), erlose (E), and sorbitol (F) concentrations of drought-susceptible (DS) and drought-tolerant (DT) wheat genotypes across the treatments [control, ABA and ACC (1-aminocyclopropane-1-carboxylic acid) spray]**.

Supplementary Table S1 **Two-way ANOVA for the effects of treatments (well-watered and mild drought) and stress group (drought-tolerant and drought-susceptible) and their interaction on several carbohydrate concentrations of six wheat genotypes that were either sprayed with water (controls), abscisic acid (ABA, 0.1 μM) or the ethylene-precursor, 1-aminocyclopropane-1-carboxylic acid (ACC, 0.1 μM) at the 3rd leaf stage**. ^*^, ^**^, ^***^ indicate *p* < 0.001, < 0.01, and < 0.05, respectively.
