# Supplementary figures and images for: Foliar Abscisic Acid-To-Ethylene Accumulation and Response Regulate Shoot Growth Sensitivity to Mild Drought in Wheat
Source: Front Plant Sci. 2016 Apr 18;7:461. doi: 10.3389/fpls.2016.00461 (PMC4834443; doi:10.3389/fpls.2016.00461)

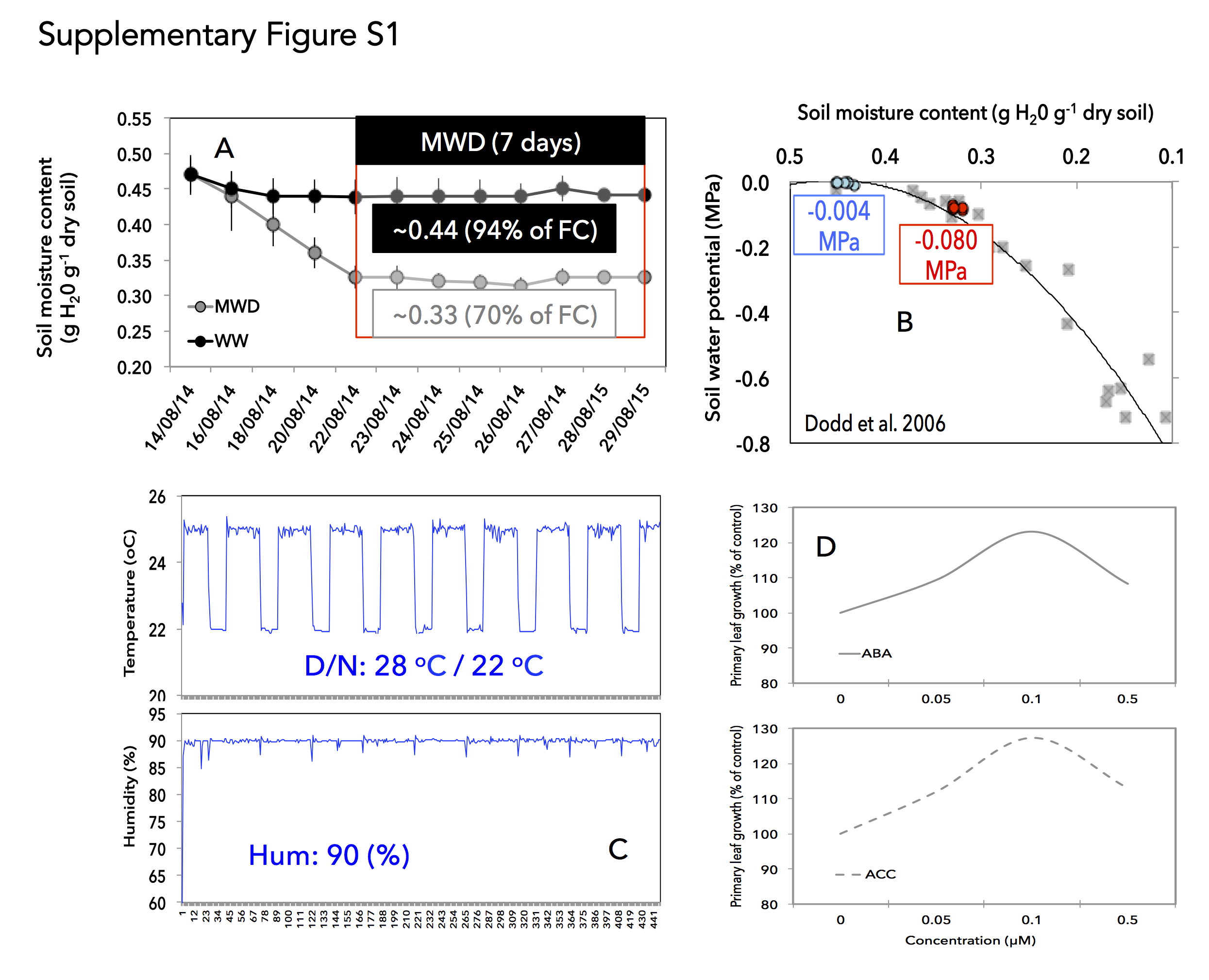

Supplement: Supplementary Figure S1 — (A), Soil moisture content (g H2O g−1 dry soil) in both well-watered and mild drought stress treatments; (B), Soil water potential (MPa) of both well-watered and mild-drought stressed plants; (C), Temperature and humidity levels during the experiment, and (D), Primary leaf growth response to different concentrations of ABA and ACC. [file Image1.JPEG]

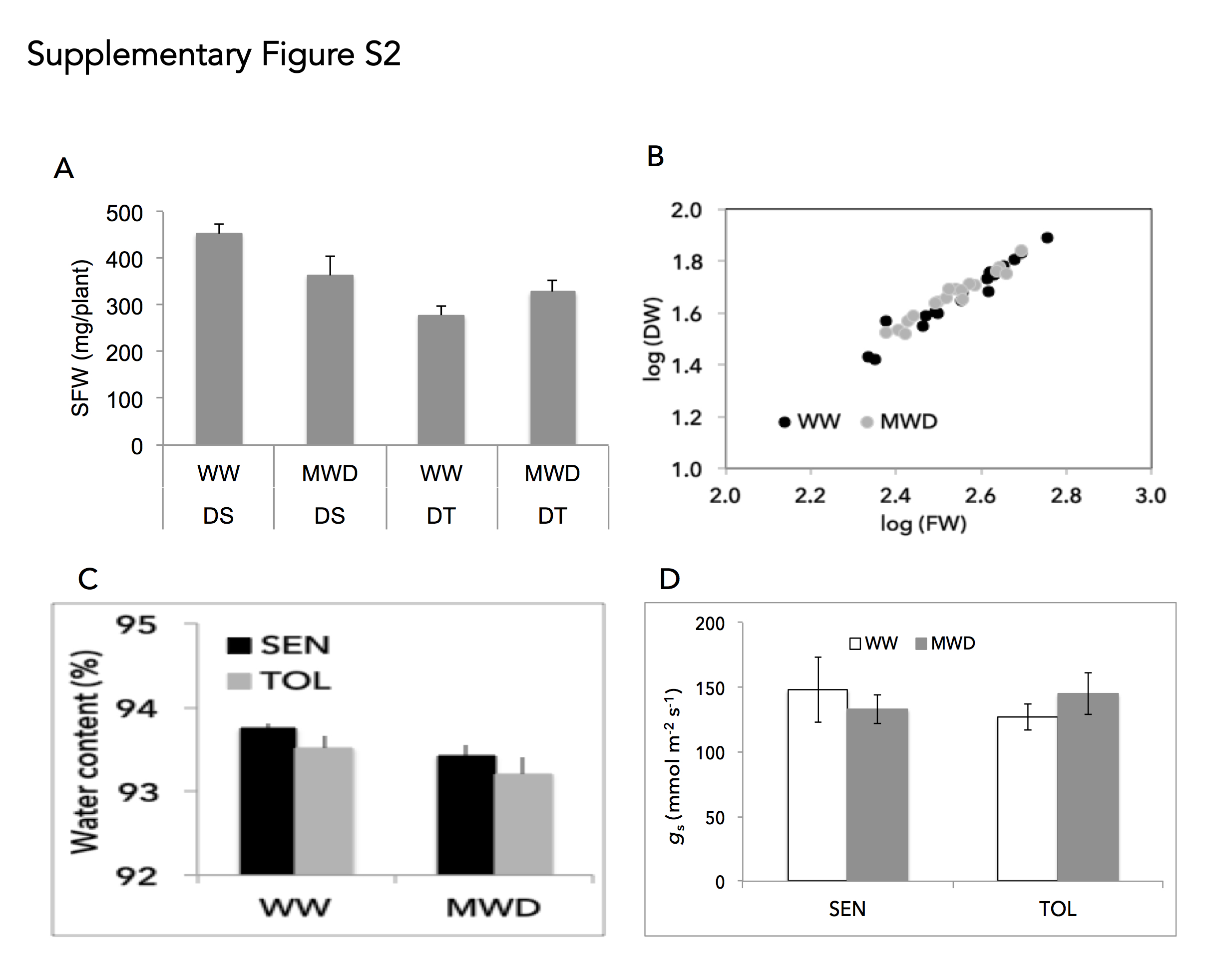

Supplement: Supplementary Figure S2 — (A) Shoot fresh weight of six wheat genotypes that were either grown under well-watered (WW) or mild water-deficit (MWD) conditions. (B), the relationship between log values of fresh weight (FW) and dry weight (DW) in WW and MWD treatments; (C), plant water content (%) of drought-susceptible and drought-tolerant wheat genotypes in WW and MWD treatments; (D), stomatal conductance of drought-susceptible and drought-tolerant wheat genotypes in WW and MWD treatments. [file Image2.JPEG]

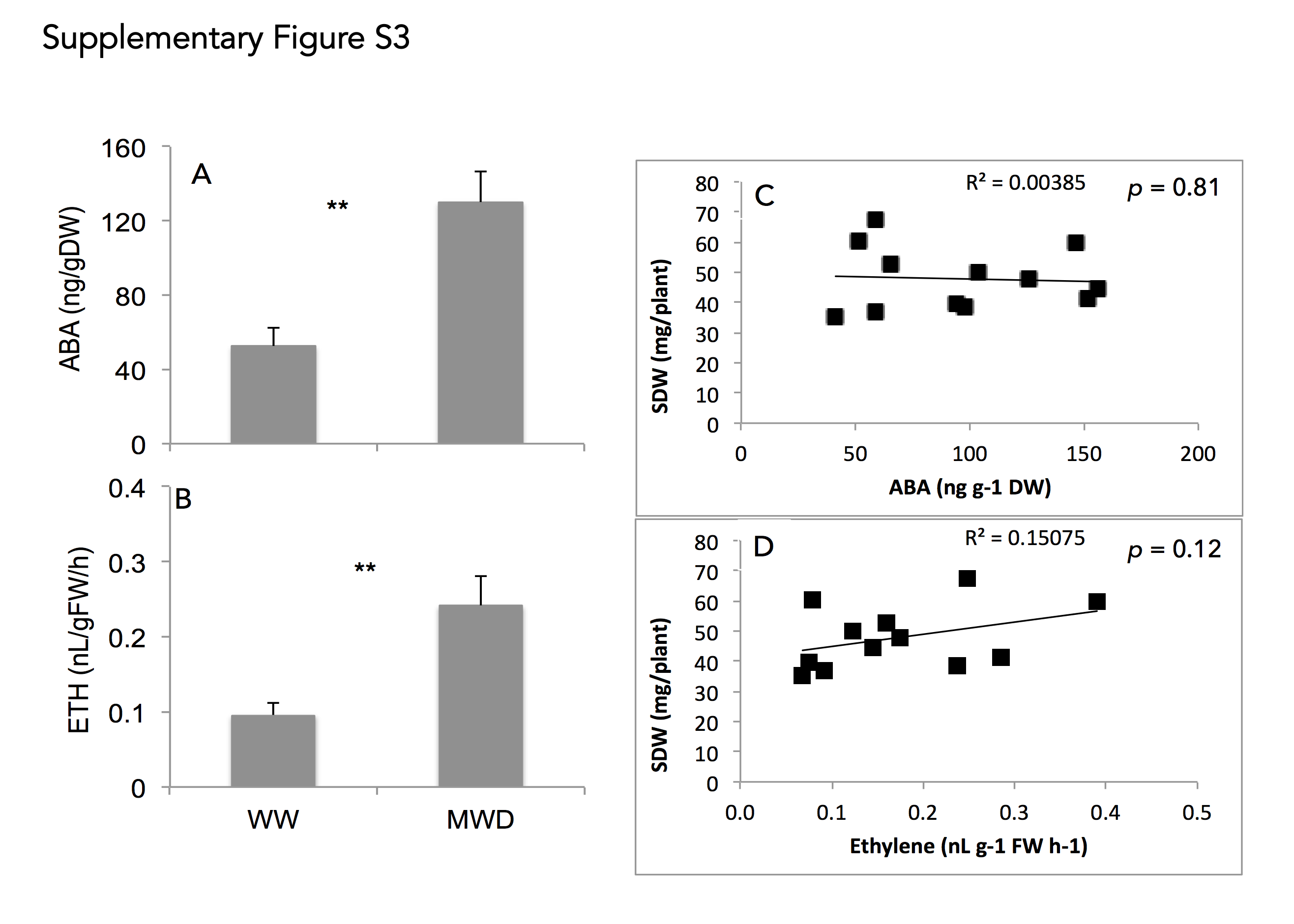

Supplement: Supplementary Figure S3 — ABA concentration (A) and ethylene evolution (B) in well-watered (WW) and mild-drought stress (MWD) treatments across all genotypes. Linear correlations between shoot dry weight (SDW) and endogenous ABA (C) and ethylene (D) across all groups and treatments. ** indicates p < 0.01. [file Image3.JPEG]

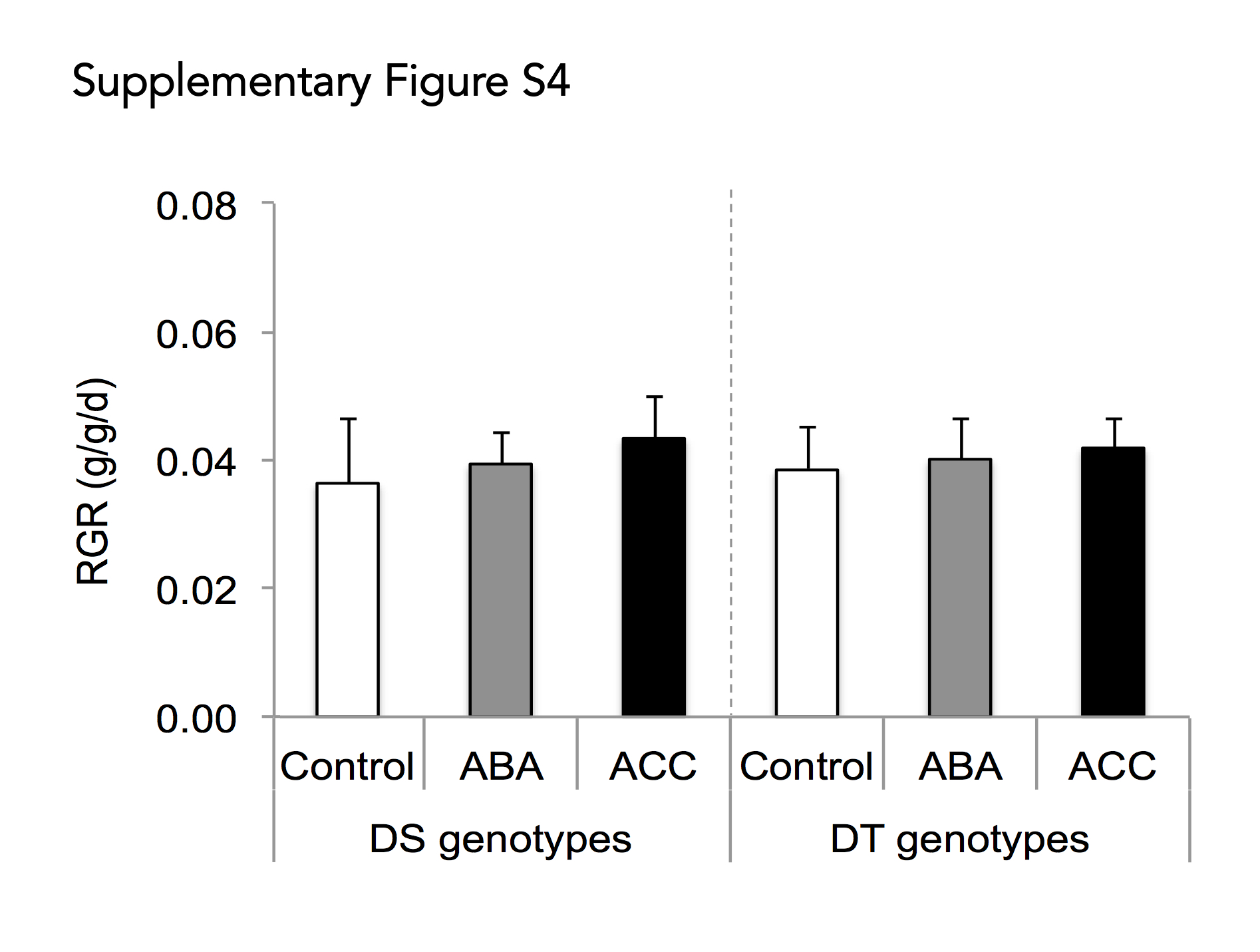

Supplement: Supplementary Figure S4 — Shoot relative growth rate in drought-susceptible (A) and drought-tolerant (B) wheat genotypes that were either sprayed with water (controls), abscisic acid (ABA, 0.1 μM) or the ethylene-precursor, 1-aminocyclopropane-1-carboxylic acid (ACC, 0.1 μM) at the 6th leaf stage. [file Image4.JPEG]

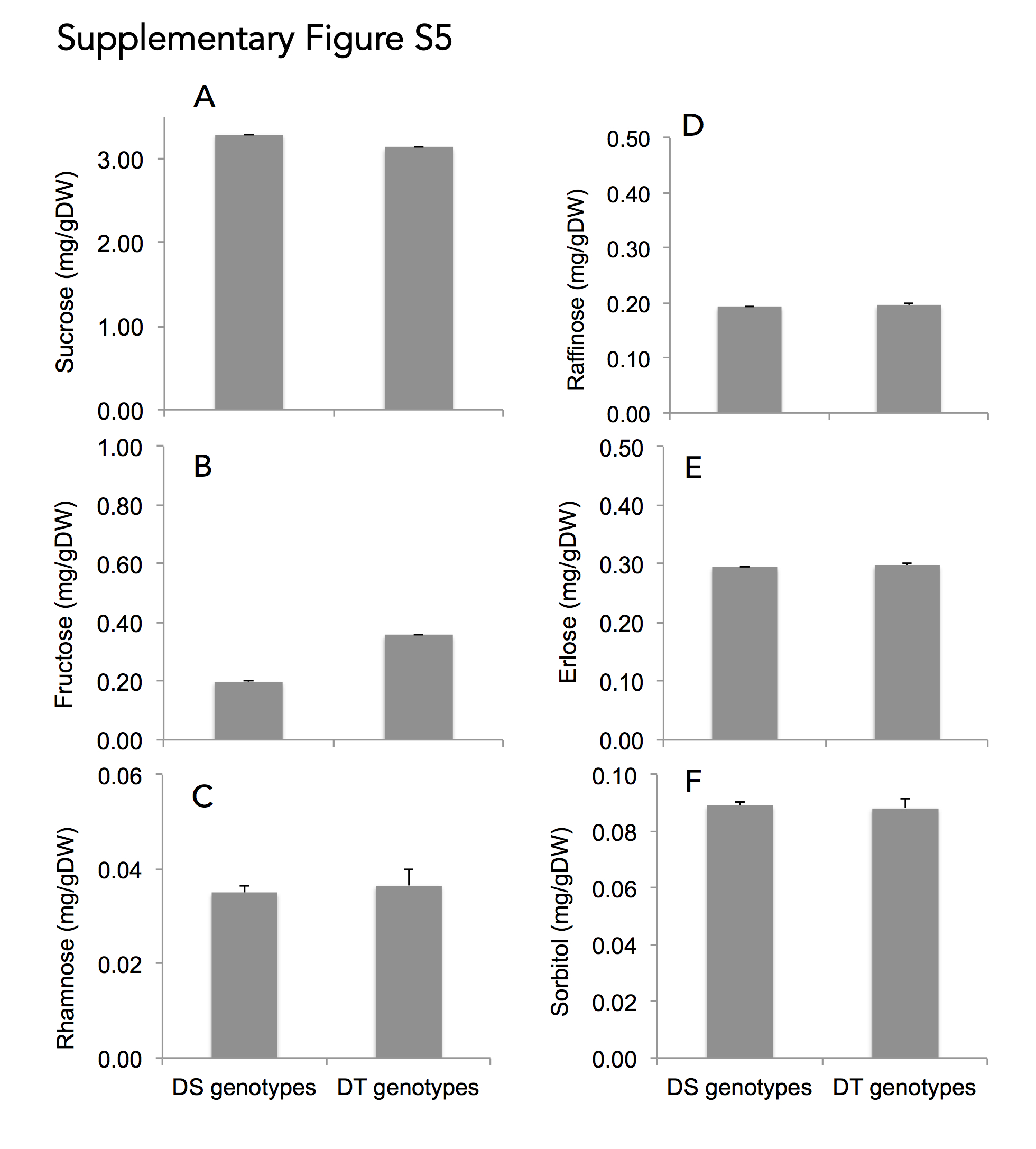

Supplement: Supplementary Figure S5 — Sucrose (A), fructose (B), rhamnose (C), raffinose (D), erlose (E), and sorbitol (F) concentrations of drought-susceptible (DS) and drought-tolerant (DT) wheat genotypes across the treatments [control, ABA and ACC (1-aminocyclopropane-1-carboxylic acid) spray]. [file Image5.JPEG]
