# Supplementary material for: Foliar Abscisic Acid-To-Ethylene Accumulation and Response Regulate Shoot Growth Sensitivity to Mild Drought in Wheat
Source: Front Plant Sci. 2016 Apr 18;7:461. doi: 10.3389/fpls.2016.00461 (PMC4834443; doi:10.3389/fpls.2016.00461)
Supplement: Supplementary Table S1 — Two-way ANOVA for the effects of treatments (well-watered and mild drought) and stress group (drought-tolerant and drought-susceptible) and their interaction on several carbohydrate concentrations of six wheat genotypes that were either sprayed with water (controls), abscisic acid (ABA, 0.1 μM) or the ethylene-precursor, 1-aminocyclopropane-1-carboxylic acid (ACC, 0.1 μM) at the 3rd leaf stage. *, **, *** indicate p < 0.001, < 0.01, and < 0.05, respectively. [file Table1.pdf]

Supplementary Table S1

Sugars	Treatment	Category	Treatment *Category
Sucrose	ns	ns	ns
Glucose	0.000***	0.000***	ns
Fructose	0.004**	ns	ns
Raffinose	ns	ns	0.003
Erlose	ns	ns	ns
Maltose	0.004**	0.000***	0.000***
Galactose	0.000***	0.000*	ns
Rhamnose	ns	ns	0.025*
Sorbitol	ns	0.000*	ns
